# Rapid Quantification of Microvessels of Three-Dimensional Blood–Brain Barrier Model Using Optical Coherence Tomography and Deep Learning Algorithm

**DOI:** 10.3390/bios13080818

**Published:** 2023-08-15

**Authors:** Huiting Zhang, Dong-Hee Kang, Marie Piantino, Daisuke Tominaga, Takashi Fujimura, Noriyuki Nakatani, J. Nicholas Taylor, Tomomi Furihata, Michiya Matsusaki, Satoshi Fujita

**Affiliations:** 1 AIST-Osaka University Advanced Photonics and Biosensing Open Innovation Laboratory, National Institute of Advanced Industrial Science and Technology (AIST), 2-1 Yamadaoka, Suita 565-0871, Osaka, Japan; zhang-huiting@aist.go.jp (H.Z.); jn.taylor@aist.go.jp (J.N.T.); m-matsus@chem.eng.osaka-u.ac.jp (M.M.); 2 Graduate School of Engineering, Osaka University, 2-1 Yamadaoka, Suita 565-0871, Osaka, Japan; metpolym@gmail.com (D.-H.K.); m-piantino@chem.eng.osaka-u.ac.jp (M.P.); 3 Cellular and Molecular Biotechnology Research Institute, National Institute of Advanced Industrial Science and Technology (AIST), 1-1-1 Higashi, Tsukuba 305-8565, Ibaraki, Japan; tominaga@my-pharm.ac.jp; 4 SCREEN Holdings Co., Ltd., 322 Furukawa-cho, Hazukashi, Fushimi-ku, Kyoto 612-8486, Kyoto, Japan; t.fujimura@screen.co.jp (T.F.); n.nakatani@screen.co.jp (N.N.); 5 School of Pharmacy, Tokyo University of Pharmacy and Life Sciences, 1432-1 Horinouchi, Hachioji 192-0392, Tokyo, Japan; tomomif@toyaku.ac.jp

**Keywords:** OCT image processing, 3D BBB model, vessel quantification

## Abstract

The blood–brain barrier (BBB) is a selective barrier that controls the transport between the blood and neural tissue features and maintains brain homeostasis to protect the central nervous system (CNS). *In vitro* models can be useful to understand the role of the BBB in disease and assess the effects of drug delivery. Recently, we reported a 3D BBB model with perfusable microvasculature in a Transwell insert. It replicates several key features of the native BBB, as it showed size-selective permeability of different molecular weights of dextran, activity of the P-glycoprotein efflux pump, and functionality of receptor-mediated transcytosis (RMT), which is the most investigated pathway for the transportation of macromolecules through endothelial cells of the BBB. For quality control and permeability evaluation in commercial use, visualization and quantification of the 3D vascular lumen structures is absolutely crucial. Here, for the first time, we report a rapid, non-invasive optical coherence tomography (OCT)-based approach to quantify the microvessel network in the 3D *in vitro* BBB model. Briefly, we successfully obtained the 3D OCT images of the BBB model and further processed the images using three strategies: morphological imaging processing (MIP), random forest machine learning using the Trainable Weka Segmentation plugin (RF-TWS), and deep learning using pix2pix cGAN. The performance of these methods was evaluated by comparing their output images with manually selected ground truth images. It suggested that deep learning performed well on object identification of OCT images and its computation results of vessel counts and surface areas were close to the ground truth results. This study not only facilitates the permeability evaluation of the BBB model but also offers a rapid, non-invasive observational and quantitative approach for the increasing number of other 3D *in vitro* models.

## 1. Introduction

The blood–brain barrier (BBB) is a semi-permeable interface between blood circulation and the extracellular fluid of the central nervous system (CNS), and its unique multicellular structures maintain brain homeostasis by tightly controlling the selective transport of molecules, ions, and other nutrients, protecting against toxins and pathogens from blood to brain [1]. The disruption of the BBB leads to multiple neurological diseases including Alzheimer’s disease, multiple sclerosis, stroke, and brain trauma [2]. To understand its role in diseases and to evaluate drug delivery to the CNS, various kinds of *in vitro* BBB models such as microfluidic, spheroid, and transwell models have been developed [3,4,5,6], providing a valuable platform for screening and evaluating drugs that can be delivered to the CNS. With the advantage of low cost, wide commercial availability, and ease of use for drug screening, transwell systems provide a significant role in BBB research [7]. Conventional BBB models are conducted by culturing brain microvascular endothelial cells (BMECs) on a transwell insert to form a monolayer for the apical side, while other types of cells such as astrocytes, pericytes, or neurons are cultured on the other side of the insert for the basal side [8,9]. Though these 2D systems exhibited high transepithelial resistance (TEER) and low permeability indicating well barrier tightness, they cannot mimic key features of the BBB perfectly. That is, all cells are grown on a flat platform where the 2D structure of cells lacks the complex extracellular matrix and cellular contacts.

Recently, we developed a 3D BBB *in vitro* model in which BMEC, pericytes, and astrocytes self-organized in fibrin gel to form a three-dimensional vascular network like natural BBB structures. BMEC formed a monolayer at the bottom of the hydrogel and fused to the vascular cells in the gel to generate a 3D vascular network with capillary opening ends during the 7-day incubation. It not only mimics the native microenvironment, which allows the three BBB cell types to directly communicate, but also offers a connection between the outside and the capillary lumen, which can be easily used for the tested molecules to perfuse from the open end inside the lumen. The model shows key features of the native BBB, such as size-selective permeability of different molecular weights of dextran, activity of P-glycoprotein efflux pump, and the functionality of receptor-mediated transcytosis (RMT) [10]. It provides a potential platform for drug screening across the BBB. To validate the integrity and functionality of the BBB model and perform drug screening in commercial use, permeabilities of specific substrates need to be checked. The effective permeability coefficient (P_e_) is generally used to characterize the transport efficiency of substrate molecules from the apical (basolateral) to basolateral (apical) side; it is generated from the permeability surface area product (PS_e_) across the endothelial barrier divided by the total surface area of endothelial cells. Therefore, knowing about the total surface area of endothelial cells is essential in the assessment of molecular permeability. In addition, the surface area of endothelial cells can reflect the dynamic generation of the vascular network, and it is also essential in TEER assessment, which is another widely used technique to assess the integrity of BBB. However, the BBB model we have developed has a three-dimensional network of vessels, which makes it difficult to calculate the surface area of the vessels as in the 2D method. Therefore, a technique for the visualization of 3D vascular architecture and quantification of the surface area of our model is crucial.

Several imaging techniques have been widely used for imaging the 3D vascular network such as using X-ray micro-computed tomography (microCT) on the vascular network perfused with a radiopaque polymer. microCT obtains high-resolution images; however, the presence of polymer in the vessel limited other analyses of tissues [11]. Other techniques such as two-photon microscopy and confocal microscopy have been used to obtain reasonably good visualization of the vessel network [12]; however, the image acquisition process of the 3D vessel structures is demanding and extremely time consuming. Additionally, these methods require fluorescence labeling using immunostaining on tissue samples. It was reported that there were changes in cells and tissue volumes after fixation [13], and the resulting vascular information may be biased from living cells. Furthermore, the fixed sample limited the dynamic analysis of the vascular evolution and permeability assessment. Second harmonic generation (SHG) and third harmonic generation (THG) microscopy are label-free imaging techniques that utilize nonlinear optical processes to visualize biological tissues, such as astrocytes and gliovascular interfaces [14], and mammalian tissue containing both skeletal muscle and fibrillar collagen [15,16]. However, THG and SHG require certain molecules that are sensitive to refractive index variations at interfaces or have non-centrosymmetric structures, respectively [16,17]. To visualize and quantify the global morphology of micro-luminal structures of the 3D transwell BBB model for quality control in commercial use, a rapid and non-invasive approach is needed. Optical coherence tomography (OCT) is a powerful imaging technology based on interference between a split and later recombined broadband optical field [18]. It is able to provide non-invasive 3D images of inhomogeneous samples and has been commonly used in the ophthalmological field [19]. Recently, OCT has been used for imaging of spheroidal BBB models [20], bovine preimplantation embryos [21], and 3D microvasculature modes [22] to visualize the dynamics of angiogenic sprouting due to the advantages of fast image acquisition, wide ranges of sample depths, and because sample preparation is unnecessary.

With the increasing application of OCT for *in vitro* models, there is a need for processing technologies to identify regions of interest (ROI) within the 3D images and derive quantitative values from OCT images for further analysis of the desired structures. However, to the best of our knowledge, there are no studies to date that have comprehensively examined the processing of OCT images of 3D *in vitro* vascular models.

**Figure 1:**
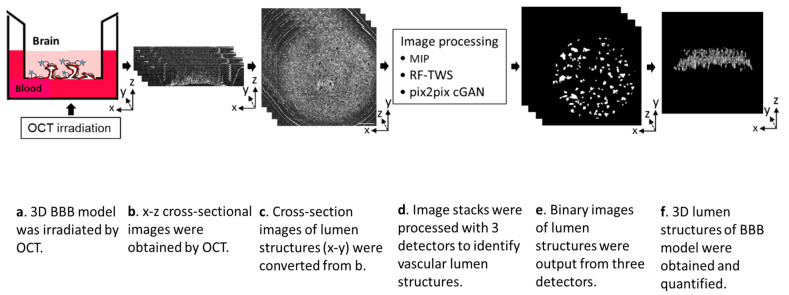
The workflow of non-invasive OCT-based approach to visualize lumen structures of 3D BBB *in vitro* model. The x-z cross-sectional images obtained from OCT were converted to z-stacks of x-y images. These converted images were analyzed with morphological imaging processing (MIP), RF machine learning using TWS plugin (RF-TWS), and deep learning using pix2pix cGAN and then the output images were used for visualization and quantification of the 3D vessel network of BBB model.

In this study, we report a rapid and non-invasive OCT-based approach to visualize and quantify the vessel lumen structures of the 3D BBB *in vitro* model. OCT was applied to visualize the vascular network of the 3D BBB model, and the resultant image stacks were processed using three detectors (morphological imaging processing (MIP), random forest machine learning using the Trainable Weka Segmentation plugin (RF-TWS), and deep learning using pix2pix cGAN) to identify vascular lumen structures. Their performances were evaluated by comparing their output images with manually selected ground truth images. Finally, the vessel counts and total surface areas of the vascular structures were calculated (Figure 1). Our approach not only facilitates the permeability assessment of the 3D BBB model but also offers a rapid and non-invasive observational and image processing approach for the increasing number of other 3D *in vitro* models.

## 2. Materials and Methods

### 2.1. Cell Culture and Fabrication of 3D BBB Model

The 3D BBB model was prepared as previously reported [10]. Briefly, human brain microvascular endothelial cells/conditionally immortalized clone 18 (HBEC) [23], human astrocyte/conditionally immortalized clone 35 (HA) [24], and human brain pericyte/conditionally immortalized clone 37 (HP) [25] were resuspended into a fibrin gel with the ratio of 2:4:1 (HBEC:HA:HP), and then the gel was polymerized in membrane-free transwell inserts to partition the apical side from the basolateral side. The next day, HBEC were seeded onto the bottom of the fibrin gel to form the HBEC monolayer, fusing to the endothelial vascular in the gel to form a luminal vascular network with perfusable opening structures after 7 days of culture (Figure 1a).

### 2.2. Image Acquisition and Preprocessing

For the OCT imaging of the *in vitro* BBB model, the samples were captured from the bottom of the fibrin gel after culturing for 7 days by using a spectral domain OCT (SD-OCT) (SCREEN Holdings Co., Ltd., Kyoto, Japan). The system was operated at a center wavelength of 890 nm, using an A-scan rate of 6000 Hz with a sensitivity of 107 dB, 10 µm for the low-resolution mode and 3 µm for the high-resolution mode. The OCT provided 2D x-z cross-sectional images (600 images) with 600 × 60 pixels (width × height; 10 µm per pixel) and 10 µm thickness (Figure 1b). For comparison with fluorescence images, the y stacking of x-z images was converted to z stacking of x-y images (60 images) (Figure 1c).

To confirm that the luminal structures visualized by OCT are microvascular structures, the samples after OCT measurement were subsequently used for fluorescence imaging on living cells. CD31 was used as the biomarker for the presence of endothelial cells that formed blood vascular lumens [26]. Samples were washed twice with phosphate-buffered saline (PBS) and incubated with Alexa Fluor^®^ 488 Anti-CD31 antibody (Abcam, ab215911, Cambridge, UK) at 37 °C for 1 h, followed by two additional PBS washes. The resultant samples were scanned by a confocal laser-scanning microscope Olympus FV3000 (Olympus, Tokyo, Japan) using a 10× objective lens with a step size of 2 µm. The scan parameters were kept the same for all of the measurements of six BBB samples.

We initially obtained 3D OCT images of each sample with depths up to 600 µm, but images were truncated to a depth of 300–350 µm based on the visible range of microvessels. The identified images were preprocessed with histogram equalization to increase the global contrast before analysis. Finally, 6 datasets obtained from 6 BBB samples were separated into 2 sets of 3 for training and testing, respectively. Each dataset contains 30–35 images, and each image had a size of 600 × 600 pixels. Three detectors (MIP, RF-TWS, and pix2pix cGAN) (Figure 1d) were applied to each testing dataset. Binary images of luminal structures output by three detectors (Figure 1e) were used to generate 3D images (Figure 1f). Unless stated otherwise, Fiji software (version 2.9.0) [27] was used for image processing.

### 2.3. Morphological Imaging Processing (MIP)

The interfacial tension between the hydrogel and the transwell insert causes a depression at the center of the hydrogel (Figure S1a); thus, the 3D OCT images are vacant in their centers for the initial few layers of the image stacks (Figure S1b). The edge of the culture-insert showing high brightness caused disturbances in the recognition of the luminal structures on binarized images. To remove these untargeted regions (edge and center vacancy of the images), we first identified the binary region of interest (ROI) mask for each image stack (Figure S2a). For main image processing, Gaussian blur and adaptive thresholding were performed on the preprocessed images and inverted for morphological operations. The adaptive threshold value was adjusted with reference to the ground truth images for correct detection and followed by morphological erosion and opening with connected component labeling to remove large and small objects, respectively. The resulting images were combined and filtered by the ROI mask, forming the binary output images (Figure S2b). This work was implemented with Python.

### 2.4. RF Machine-Learning Using TWS (RF-TWS)

The semi-automatic Trainable Weka Segmentation 3D (TWS) [28] is a robust and user-friendly Fiji plugin of ImageJ, containing a large number of machine learning algorithms for classifier training, and the random forest (RF) algorithm was used for this study. In detail, the stack images from the training dataset were input into the classifier, and the representative pixels of the vascular lumen and background of each slice were randomly selected and labeled as two classes for training. Once the classifier is obtained, the remaining unlabeled pixels are classified, and binary classification images are output. Classification results are inferred from the binary images, and then misclassifications are corrected manually before repeatedly retraining the classifier and correcting misclassifications using different randomly selected subsets to obtain more accurate results. Final output images were post-processed with morphological closing to remove small, unwanted objects (Figure S3).

### 2.5. Deep Learning Using Pix2pix cGAN

As a supervised image-to-image solution, the Pix2pix conditional generative advertise network (Pix2pix cGAN) containing a U-Net generator plus a PatchGAN discriminator [29] does not need to know about specific features of input images but learns the differentiation of both classes through the simultaneous training of adversarial discriminative and generative networks. In our case, 104 OCT image frames from three individual datasets were horizontally and vertically flipped for training (training datasets 1–3), while 101 OCT image frames from the other three datasets were used for testing (testing datasets 1–3). For classifier training, we used a patch-based strategy to learn discriminative features. Briefly, each of the input images was split into nine overlapping 256 × 256-pixel patches, and they were fed to the generator and translated to the target (output) images (Figure S4). This work was implemented with Mathematica.

### 2.6. Performance Evaluation

In this study, the manually labeled binary images derived from testing datasets 1–3 served as the ground truth. Agreement between the ground truth images and those of different image processing methods was evaluated with the inter-rater reliability measure known as Cohen’s kappa [30].

(1) $$\kappa =\frac{\left(TP\times TN\right)-\left(FP\times FN\right)}{\left(TP+FP\right)\times \left(FP+TN\right)+\left(TP+FN\right)\times \left(FN+TN\right)}$$

where TP, FP, TN, and FN represent the numbers of true positives, false positives, true negatives, and false negatives in an image frame, respectively. A segmented object pixel that overlapped with ground truth is considered a true positive; otherwise, it is a false positive. The positive ground truth that has no pixels overlapped by a segmented object is counted as a false negative, while a negative ground truth that does not overlap a segmented object is considered a true negative.

## 3. Results and Discussion

### 3.1. Visualization of Microvascular Structures in BBB Model by OCT

Typically, confocal microscopy (CM) is very effective for visualizing 3D tissue model structures *in vitro*, whereas limitations such as light scattering and numerical aperture (NA) of the objective limited the penetration depth to less than 100 µm [31]. In addition, to acquire images of 3D tissue samples with CM, the tissue samples need to be preprocessed with immunostaining, which is time consuming (more than 2 days), and scanning of the large volume (8457 × 8382 × 180 µm^3^) of the BBB model is demanding, requiring a complicated image stitching process to combine 49 smaller 3D images (1208 × 1197 × 180 µm^3^) into reconstructed 3D global images of our BBB tissue sample. On the other hand, since OCT typically has higher transmission optics but lower resolution than confocal microscopy, we expect that OCT can visualize deeper vascular structures of our 3D BBB tissue in shorter times. Therefore, we first evaluated the potential of OCT imaging for visualizing the global microvascular lumen morphology of the BBB model.

**Figure 2:**
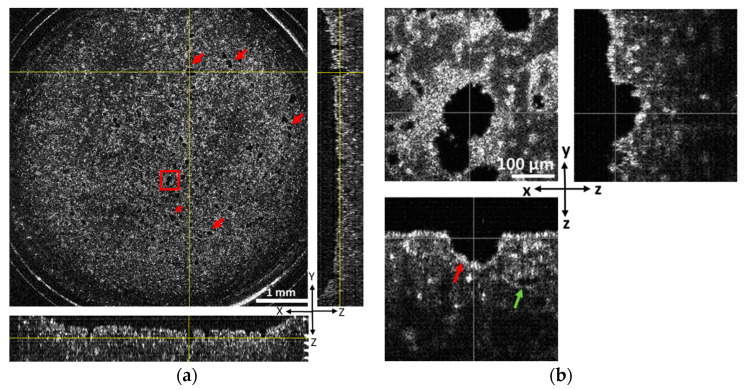
Orthogonal views (xy, xz, and yz) of the converted OCT images showing 3D structure of microvessels in the BBB model. (**a**). Top, front, and side views of a representative OCT image located at 160 µm depth from bottom side of the tissue structure. The lengths of the *x*-axis, *y*-axis, and *z*-axis are 6000, 6000, and 900 µm, respectively (z was magnified 3 times), and vessel lumen structures are indicated with red arrows. (**b**). The high-magnification images of the red boxed area of a and open and closed vascular structures are indicated with red and green arrows, respectively. Scale bar of the global and magnification image is 1 mm and 0.1 mm, respectively.

Our BBB model sample consists of a fibrin hydrogel construct that is ~1200 µm thick with the endothelial micro-tubular lumina extending from its bottom surface, which is covered with a monolayer of endothelial cells (Figure 1a). Numerous pore-like morphologies representing the cross-sectional lumen area are obvious in the representative OCT image, which is located at a 160 µm depth from bottom side of the construct (Figure 2a). In addition, we also observed closed micro-luminal structures inside the gel (Figure 2b and Figure S6). Open and closed luminal structures could be observed more clearly with the high-magnification mode of OCT (Figure 2b). Although OCT could capture the structures from its bottom side up to a depth of 600 µm, clear images could not be obtained beyond a 300–350 µm depth due to the gradual blurring of OCT images. Because open micro-luminal structures connected to the bottom side rarely extended beyond a depth of 350 µm, there is no major problem for our purpose such as permeability assessment, even if other closed luminal structures may be present.

The sample was subsequently stained with the endothelial marker CD31 and observed by confocal microscopy to confirm that the luminal structures visualized by OCT are microvascular structures. Although the two images cannot be perfectly matched due to totally different measurement principles, image resolutions, etc., between the OCT and CM, the matched objects in both images can demonstrate that the pore-like morphology in the OCT image is micro-vascular lumens formed by endothelial cells (Figure S5), as almost all micro-luminal structures are identical to microvessel structures. Moreover, we successfully obtained full 3D images of our BBB model sample with the volume of 6000 × 6000 × 600 µm^3^ (after conversion) in only 14 min using the OCT method, therefore confirming that OCT imaging is a powerful tool for visualizing micro-luminal structure including microvessels in our BBB model.

### 3.2. Image-Based Evaluation of Performance of Three Detectors

In order to quantify the vascular lumen structure of the 3D BBB *in vitro* model, it is necessary to accurately and selectively/specifically extract the vascular lumen structure from the OCT images obtained. However, OCT images generally lose resolution as the depth of the tissue increases and OCT images of the tissue model change in appearance. Therefore, it is challenging to extract the pore-like morphology precisely from 3D image stacks including some images with low contrast or inevitable noise. In our study, we initially obtained 3D images of each sample with depths up to 600 µm, but it was deemed that most open structures extended less than 300–350 in depth; images were cropped to this depth to avoid resolution and noise issues as much as possible. Then, the performance of three potential detectors (MIP, RF-TWS, and Pix2pix cGAN) in vascular lumen recognition was compared on 3D OCT images derived from one BBB model.

**Figure 3:**
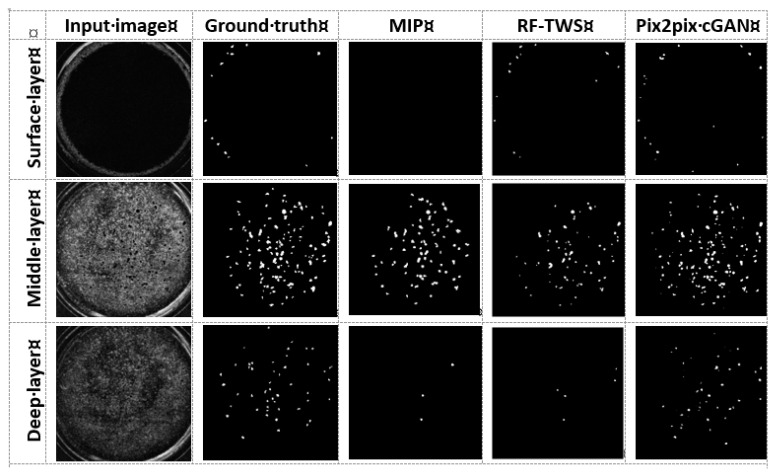
Input and output images identified by manual selection (ground truth), morphological imaging processing (MIP), Random forest machine learning using Trainable Weka Segmentation plugin (RF-TWS), and deep learning using pix2pix cGAN (pix2pix cGAN) of surface (0~50 µm), middle (50~200 µm), and deep layer (200~310 µm) images.

First, the 31 images in testing dataset 1 acquired by OCT were segregated into three regions according to the *z*-axis direction showing the different morphologies. Each representative image is shown on the left side of Figure 3. The images of the surface region, including layers 1~5, had vacancies in the center due to the central hydrogel depression caused by the interfacial tension between the hydrogel and culture insert, whereas tissue and luminal structures were observed in the region at the edge of the insert (top-left). The images of the middle region, including layers 6~20, showed a relatively clear contrast between tissue and luminal structures (middle-left). The images of the deeper region, including layers 21~31, gradually became unclear due to the transparency of OCT (bottom-left). These original OCT images were processed by three detectors as input images, and, as a control, the luminal structure was manually marked and used as ground truth images to evaluate each detector.

In the detector based on morphological imaging processing, we manually tried to recognize the luminal structures by a combination of general processes including Gaussian blur, adaptive thresholding, and morphological erosion/opening using testing datasets 1, 2, and 3. Edges of the culture-insert showing high brightness caused disturbances in the recognition of the luminal structures using MIP methods on binarized images. Therefore, a circular ROI mask with r = 250 pixels was first created and implemented through all stacks to remove the interference of the insert edge, and finally, the parameters of each process for classifying luminal structures and others were determined. However, despite the manual optimization of each parameter as much as possible, recognition of the luminal objects at the edges of the culture insert was unsuccessful. As a result, few luminal structures were detected in some layers belonging to the surficial region (top-third from left). On the other hand, in comparison to the ground truth images, objects at the middle depths were well recognized with the MIP detector, although recognition accuracy in deeper regions is poor (middle-third from the left and bottom-third from the left).

Initially, two types of detectors, RF-TWS and Pix2pix cGAN, were trained using three datasets (training datasets 1, 2, and 3) and validated using the other three datasets (testing datasets 1, 2, and 3). However, the classifier from training datasets was unsuccessful on the test datasets when using RF-TWS. Thus, we directly applied the RF-TWS detector to the testing images to identify luminal structures. As a result, the locations of many objects were successfully detected without ROI mask application at layers belonging to the surficial (top-center and top-right) and middle regions (middle-center and middle-right). Similar to the MIP detector, the RF-TWS detector shows poor object recognition at larger depths, possibly caused by images with lower contrast and uneven pixel intensity distributions. Notably, Pix2pix cGAN identified most of the objects even with low contrast and complex background (bottom-center).

### 3.3. Quantitative Evaluation of the Performance of 3 Detectors

**Figure 4:**
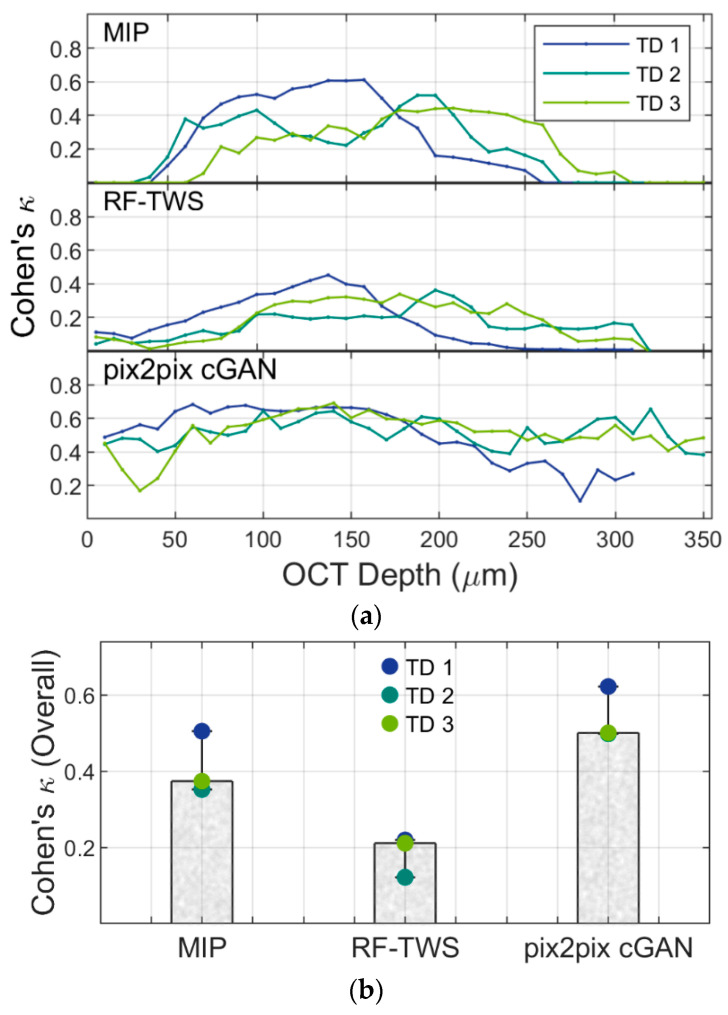
(**a**). Cohen’s kappa coefficient (Equation (1)) of each frame with increasing depths (from left to right) of three datasets; each frame was 10 µm depth. (**b**). Averaged Cohen’s *κ* of three approaches calculated from test datasets 1, 2, and 3; error bar represents maximum and minimum value.

Next, for a more detailed assessment, we evaluated the performance of three detectors quantitatively using three testing datasets. The output binary images showing locations of luminal structures located by three detectors at each depth layer in each dataset were compared with ground truth images using Cohen’s *κ* coefficient (Equation (1)) to evaluate the recognition accuracy of luminal structures (Figure 4). Cohen’s *κ* coefficient measures the level of agreement between two judges; in our case, they are the corresponding pixels of output images and the ground truth images. The coefficient can range from −1 to +1, and larger values of *κ* are better. It is suggested that values from 0.21 to 0.40 are fair, from 0.41 to 0.6 are moderate, and from 0.61 to 0.8 show substantial agreement [30,32].

The three image datasets (testing datasets 1, 2, and 3), which consist of 31, 35, and 35 z-stack images, respectively, were captured from three individual BBB samples. The contrast and background of the images were slightly different in the respective datasets due to lot-to-lot differences in cell proliferation and formation of luminal structures in fibrin hydrogel constructs. As a result, the images in testing dataset 1 were more easily detected compared to the other two datasets. For the MIP detector, parameters were manually optimized to process the images of the three testing datasets, but with a low detection rate, indicated by values of *κ* between 0.2~0.4. Test dataset 1, however, showed higher detection performance than the other two, with *κ* ranging from 0.4 to 0.6 in the depth range of 80 µm to 200 µm (Figure 4a, upper). For RF-TWS, almost all *κ*-values were less than 0.4 in the stack images of the three image datasets. The result shows that trained classifiers do not work well, even though the classifier was continuously optimized with its powerful interface feedback. This may be due to the inconsistency of the three datasets and the strong noise in OCT images, causing the classifier to misidentify many noise signals in the vacant areas of the image as hole-like structures in the images (Figure 4a, middle). For the deep-learning-based detector, the *κ* values were above 0.6 for the 100~200 µm depth range and 0.4~0.6 for depth ranges of 0~100 µm and 200 µm~350 µm. The results show sufficient accuracy, and the detector performed with good consistency and reproducibility in the three testing datasets (Figure 4a, bottom).

The averaged *κ* values reached nearly 0.6 using the deep-learning-based detector (Figure 4b), indicating the good prediction ability of the detector. It is suggested that deep learning generally requires large amounts of data for optimizing classifiers [33], but our classified detector, trained using 104 images derived from just three training datasets, worked well on the other three testing datasets. These results suggest that the cGANs used in our detection work have the potential to learn from limited information [34]. Patch-based methods and geometric transformations may be helpful for deep learning with limited data, and preprocessing operations such as histogram equalization might be applicable in learning models with non-uniform and low-contrast images. Overall, recognition performance using our optimized detector based on deep learning shows good agreement with the ground truth and reduces the complexity for object identification.

We investigated the performance of the three detectors via image-based and quantitative evaluation. There are advantages and disadvantages of 3D OCT image analysis with different approaches. Morphological imaging processing has good detection ability on high-contrast images with high accuracy, but it requires the development of convolutional matrices manually to realize edge detection and feature extraction of the images. The RF-TWS has a graphical user-friendly interface; it provides feedback to users after segmentation, which allows one to correct or add new labels and train again until the ideal results are obtained. However, it might not work well on inhomogeneous datasets. Both detectors above showed poor detection capability in low-contrast images. Deep-learning-based Pix2pix cGAN can automatically detect objects with the robust convolutional neural network; it performs well on both high- and low-contrast images and offers the possibility to detect objects at large scale without additional effort. However, it must be trained using a particular set of images.

### 3.4. Quantification of 3D Vascular Structures of BBB Model

The accurate quantification of vascular lumen structures of the 3D BBB model is crucial for quality control in commercial use. By measuring the vascular surface area, permeability can be assessed for further drug screening. Thus, we characterized the microvessels of our model using the output images of the deep learning algorithm and compared its results with those from ground truth images for further performance evaluation.

**Figure 5:**
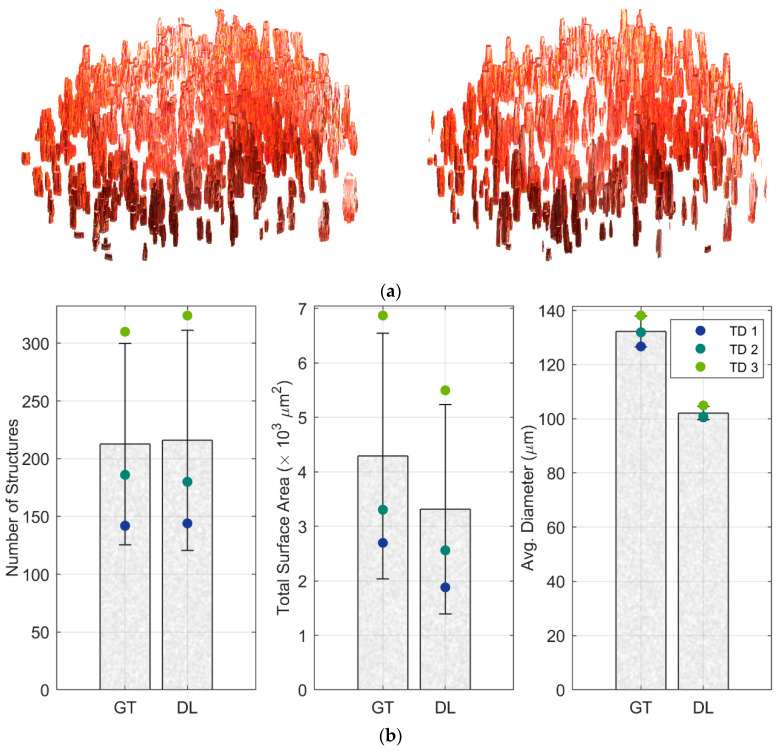
Vascular structures generated by the binary images of ground truth (**upper left**) and deep learning using pix2pix cGAN (**upper right**) of test dataset 1 (the dimension of x, y, and *z*-axis are 600, 600, and 20 pixels, respectively) (**a**), and averaged computation results (*n* = 3) of each 3D BBB model (**b**): total vessel number (**left**), total surface area of open vascular structures (**middle**) and mean diameter of each vessel (**right**) calculated from images of ground truth (GT) and deep learning (DL); error bars represent standard deviation of three datasets. *p*-values for paired *t*-tests for equivalence between GT and DL are 0.97 for the number of structures, 0.59 for the total surface area, and 0.01 for the diameters.

The 3D vascular structures were segmented from binarized images using pixel connectivity in the Image Processing Toolbox of MATLAB R2022b (Figure 5a). Since for permeability assays, the tested compounds can only perfuse from the open end inside the capillary, we define the luminal structures that are connected to the bottom surface of the fibrin gel as open vascular structures. Otherwise, structures are considered to be closed (Figure S6). Here, we removed closed vascular structures and evaluated only those open vascular structures that were involved in the permeability assay. The number of structures of each dataset was counted, the average diameter was calculated as the minimum x-y distance across each ROI object, and the total surface area was the product of object circumference in each frame and the number of frames along the *z*-axis. The number of structures predicted by deep learning is almost the same as that of the ground truth (*p* = 0.97), and values of average diameter for each microvessel (*p* = 0.01) and total surface area of each BBB model (*p* = 0.59) are close in value (Figure 5b). Though diameters returned by DL are smaller due to noise in the OCT measurement, these results demonstrate the possibility of quantifying the vascular lumen of the BBB model with OCT. In the future, deep learning automatic prediction may be suitable for both commercial and research purposes for rapid quantification of the 3D vascular open structures of the BBB.

## 4. Conclusions

For the first time, we reported a rapid approach to quantify microvessels of a 3D blood–brain barrier model using a non-invasive OCT technique to acquire 3D global image acquisition (14 min, including tomography computation) and deep learning-based image processing (about 5 h for training a classifier). However, this study has its limitations; for example, the ground truth images that were manually selected can be affected by the quality of the images, especially for the images in deep layers, and affect the evaluation accuracy. Although the detectable depth that OCT can reach is limited to 350 µm, for the assessment of 3D BBB vascular permeability, only open vascular structures are effective, and they are rarely extended beyond 300 µm depth. Structures in deeper layers are thus not required for this purpose.

Many excellent techniques can be used to study 3D *in vitro* models. Each has its own advantages and limitations, and the appropriate technique should be selected depending on the application. OCT can collect a large area of global three-dimensional images of living cell samples in a relatively short time without any sample preprocessing and can be used to visualize the dynamic generation of the vascular network. Combined with deep-learning-based image processing, morphological properties of the vessel network such as total surface area (A) can be obtained, and combined with knowledge of the permeability surface area product (PSe) of the compounds between the apical and basolateral side, the effective permeability coefficient (Pe) can be evaluated (Pe = PSe/A). Thus, vascular responses to different test molecules can be monitored in real time. The combination of OCT and deep learning provides reliable technical support for the study of 3D microvascular networks and other increasingly developed 3D models. We believe that with the rapidly growing demand for *in vitro* 3D models, this combination will be a novel way forward for characterization analysis.

## Data Availability

The data presented in this study are available on request from the corresponding author.

## Supplementary Materials

The following supporting information can be downloaded at: https://www.mdpi.com/article/10.3390/bios13080818/s1, Figure S1: View of OCT images; Figure S2: Flowchart of morphological imaging processing; Figure S3: Workflow of image processing using RF-TWS; Figure S4: Deep learning using pix2pix cGAN architecture for image processing; Figure S5: Cross-sectional view image frame obtained by OCT and confocal microscopy; Figure S6: Front view of the open vascular and closed vascular structures.

## References

[1] Obermeier B., Daneman R., Ransohoff R.M. (2013). Development, Maintenance and Disruption of the Blood-Brain Barrier. Nat. Med..

[2] Rosenberg G.A. (2012). Neurological Diseases in Relation to the Blood–Brain Barrier. J. Cereb. Blood Flow Metab..

[3] Stone N.L., England T.J., O’Sullivan S.E. (2019). A Novel Transwell Blood Brain Barrier Model Using Primary Human Cells. Front. Cell. Neurosci..

[4] Qi D., Wu S., Lin H., Kuss M.A., Lei Y., Krasnoslobodtsev A., Ahmed S., Zhang C., Kim H.J., Jiang P. (2018). Establishment of a Human IPSC- and Nanofiber-Based Microphysiological Blood–Brain Barrier System. ACS Appl. Mater. Interfaces.

[5] Nzou G., Wicks R.T., Wicks E.E., Seale S.A., Sane C.H., Chen A., Murphy S.V., Jackson J.D., Atala A.J. (2018). Human Cortex Spheroid with a Functional Blood Brain Barrier for High-Throughput Neurotoxicity Screening and Disease Modeling. Sci. Rep..

[6] Blanchard J.W., Bula M., Davila-Velderrain J., Akay L.A., Zhu L., Frank A., Victor M.B., Bonner J.M., Mathys H., Lin Y.-T. (2020). Reconstruction of the Human Blood–Brain Barrier *In vitro* Reveals a Pathogenic Mechanism of APOE4 in Pericytes. Nat. Med..

[7] Yan L., Moriarty R.A., Stroka K.M. (2021). Recent Progress and New Challenges in Modeling of Human Pluripotent Stem Cell-Derived Blood-Brain Barrier. Theranostics.

[8] Nakagawa S., Deli M.A., Nakao S., Honda M., Hayashi K., Nakaoke R., Kataoka Y., Niwa M. (2007). Pericytes from Brain Microvessels Strengthen the Barrier Integrity in Primary Cultures of Rat Brain Endothelial Cells. Cell Mol. Neurobiol..

[9] Hatherell K., Couraud P.-O., Romero I.A., Weksler B., Pilkington G.J. (2011). Development of a Three-Dimensional, All-Human *In Vitro* Model of the Blood–Brain Barrier Using Mono-, Co-, and Tri-Cultivation Transwell Models. J. Neurosci. Methods.

[10] Piantino M., Kang D.-H., Furihata T., Nakatani N., Kitamura K., Shigemoto-Mogami Y., Sato K., Matsusaki M. (2022). Development of a Three-Dimensional Blood-Brain Barrier Network with Opening Capillary Structures for Drug Transport Screening Assays. Mater. Today Bio.

[11] Ghanavati S., Yu L.X., Lerch J.P., Sled J.G. (2014). A Perfusion Procedure for Imaging of the Mouse Cerebral Vasculature by X-ray Micro-CT. J. Neurosci. Methods.

[12] Davalos D., Lee J.K., Smith W.B., Brinkman B., Ellisman M.H., Zheng B., Akassoglou K. (2008). Stable *In Vivo* Imaging of Densely Populated Glia, Axons and Blood Vessels in the Mouse Spinal Cord Using Two-Photon Microscopy. J. Neurosci. Methods.

[13] Su J.-W., Hsu W.-C., Tjiu J.-W., Chiang C.-P., Huang C.-W., Sung K.-B. (2014). Investigation of Influences of the Paraformaldehyde Fixation and Paraffin Embedding Removal Process on Refractive Indices and Scattering Properties of Epithelial Cells. J. Biomed. Opt..

[14] Lanin A.A., Pochechuev M.S., Chebotarev A.S., Kelmanson I.V., Belousov V.V., Zheltikov A.M. (2019). Nonlinear-optical Stain-free Stereoimaging of Astrocytes and Gliovascular Interfaces. J. Biophotonics.

[15] Psilodimitrakopoulos S., Artigas D., Soria G., Amat-Roldan I., Planas A.M., Loza-Alvarez P. (2009). Quantitative Discrimination between Endogenous SHG Sources in Mammalian Tissue, Based on Their Polarization Response. Opt. Express.

[16] Weigelin B., Bakker G.-J., Friedl P. (2016). Third Harmonic Generation Microscopy of Cells and Tissue Organization. J. Cell Sci..

[17] Borile G., Sandrin D., Filippi A., Anderson K.I., Romanato F. (2021). Label-Free Multiphoton Microscopy: Much more than Fancy Images. Int. J. Mol. Sci..

[18] Tomlins P.H., Wang R.K. (2005). Theory, Developments and Applications of Optical Coherence Tomography. J. Phys. D Appl. Phys..

[19] Wojtkowski M., Leitgeb R., Kowalczyk A., Bajraszewski T., Fercher A.F. (2002). *In Vivo* Human Retinal Imaging by Fourier Domain Optical Coherence Tomography. J. Biomed. Opt..

[20] Kitamura K., Umehara K., Ito R., Yamaura Y., Komori T., Morio H., Akita H., Furihata T. (2021). Development, Characterization and Potential Applications of a Multicellular Spheroidal Human Blood–Brain Barrier Model Integrating Three Conditionally Immortalized Cell Lines. Biol. Pharm. Bull..

[21] Masuda Y., Hasebe R., Kuromi Y., Kobayashi M., Urataki K., Hishinuma M., Ohbayashi T., Nishimura R. (2021). Three-Dimensional Live Imaging of Bovine Preimplantation Embryos: A New Method for IVF Embryo Evaluation. Front. Vet. Sci..

[22] Takahashi H., Kato K., Ueyama K., Kobayashi M., Baik G., Yukawa Y., Suehiro J., Matsunaga Y.T. (2017). Visualizing Dynamics of Angiogenic Sprouting from a Three-Dimensional Microvasculature Model Using Stage-Top Optical Coherence Tomography. Sci. Rep..

[23] Ito R., Umehara K., Suzuki S., Kitamura K., Nunoya K., Yamaura Y., Imawaka H., Izumi S., Wakayama N., Komori T. (2019). A Human Immortalized Cell-Based Blood–Brain Barrier Triculture Model: Development and Characterization as a Promising Tool for Drug−Brain Permeability Studies. Mol. Pharm..

[24] Furihata T., Ito R., Kamiichi A., Saito K., Chiba K. (2016). Establishment and Characterization of a New Conditionally Immortalized Human Astrocyte Cell Line. J. Neurochem..

[25] Umehara K., Sun Y., Hiura S., Hamada K., Itoh M., Kitamura K., Oshima M., Iwama A., Saito K., Anzai N. (2018). A New Conditionally Immortalized Human Fetal Brain Pericyte Cell Line: Establishment and Functional Characterization as a Promising Tool for Human Brain Pericyte Studies. Mol. Neurobiol..

[26] Albelda S.M., Oliver P.D., Romer L.H., Buck C.A. (1990). EndoCAM: A Novel Endothelial Cell-Cell Adhesion Molecule. J. Cell Biol..

[27] Schindelin J., Arganda-Carreras I., Frise E., Kaynig V., Longair M., Pietzsch T., Preibisch S., Rueden C., Saalfeld S., Schmid B. (2012). Fiji: An Open-Source Platform for Biological-Image Analysis. Nat. Methods.

[28] Arganda-Carreras I., Kaynig V., Schindelin J., Cardona A., Seung H.S. (2017). Trainable Weka Segmentation: A Machine Learning Tool for Microscopy Image Segmentation. Bioinformatics.

[29] Isola P., Zhu J.-Y., Zhou T., Efros A.A. (2018). Image-to-Image Translation with Conditional Adversarial Networks. arXiv.

[30] McHugh M.L. (2012). Interrater Reliability: The Kappa Statistic. Biochem. Med..

[31] Smithpeter C.L., Dunn A.K., Welch A.J., Richards-Kortum R. (1998). Penetration Depth Limits of *In Vivo* Confocal Reflectance Imaging. Appl. Opt..

[32] Stemler S.E. (2004). A Comparison of Consensus, Consistency, and Measurement Approaches to Estimating Interrater Reliability. Pract. Assess. Res. Eval..

[33] Sarker I.H. (2021). Deep Learning: A Comprehensive Overview on Techniques, Taxonomy, Applications and Research Directions. SN Comput. Sci..

[34] Karras T., Laine S., Aittala M., Hellsten J., Lehtinen J., Aila T. (2020). Training Generative Adversarial Networks with Limited Data. Adv. Neural Inf. Process. Syst..

