# Supplementary material for: Rapid Quantification of Microvessels of Three-Dimensional Blood–Brain Barrier Model Using Optical Coherence Tomography and Deep Learning Algorithm
Source: Biosensors (Basel). 2023 Aug 15;13(8):818. doi: 10.3390/bios13080818 (PMC10452445; doi:10.3390/bios13080818)
Supplement: Supplementary file 1 [file biosensors-13-00818-s001.zip › biosensors-2480454-supplementary.pdf]

Article

Rapid Quantification of Microvessels of Three-Dimensional Blood-Brain Barrier Model Using Optical Coherence Tomography and Deep Learning Algorithm.

Huiting Zhang [1,2], Dong-Hee Kang [2], Marie Piantino [2], Daisuke Tominaga [3], Takashi Fujimura [4], Noriyuki Nakatani [4], J. Nicholas Taylor [1], Tomomi Furihata [5], Michiya Matsusaki [1,2] and Satoshi Fujita [1,2*]

[1] AIST-Osaka University Advanced Photonics and Biosensing Open Innovation Laboratory, National Institute of Advanced Industrial Science and Technology (AIST), 2-1 Yamadaoka, Suita, Osaka 565-0871, Japan; zhang-huiting@aist.go.jp (H.Z.); jn.taylor@aist.go.jp (J.N.T.); m-matsus@chem.eng.osaka-u.ac.jp (M.M.)
[2] Graduate School of Engineering, Osaka University, 2-1 Yamadaoka, Suita, Osaka 565-0871, Japan; metpolym@gmail.com (D.-H.K.); m-piantino@chem.eng.osaka-u.ac.jp (M.P.)
[3] Cellular and Molecular Biotechnology Research Institute, National Institute of Advanced Industrial Science and Technology (AIST), 1-1-1 Higashi, Tsukuba, Ibaraki 305-8565, Japan; tominaga@my-pharm.ac.jp
[4] SCREEN Holdings Co., Ltd., 322 Furukawa-cho, Hazukashi, Fushimi-ku, Kyoto 612-8486, Japan; t.fujimura@screen.co.jp (T.F.); n.nakatani@screen.co.jp (N.N.)
[5] School of Pharmacy, Tokyo University of Pharmacy and Life Sciences, 1432-1 Horinouchi, Hachioji, Tokyo 192-0392, Japan; tomomif@toyaku.ac.jp
* Correspondence : s-fujita@aist.go.jp

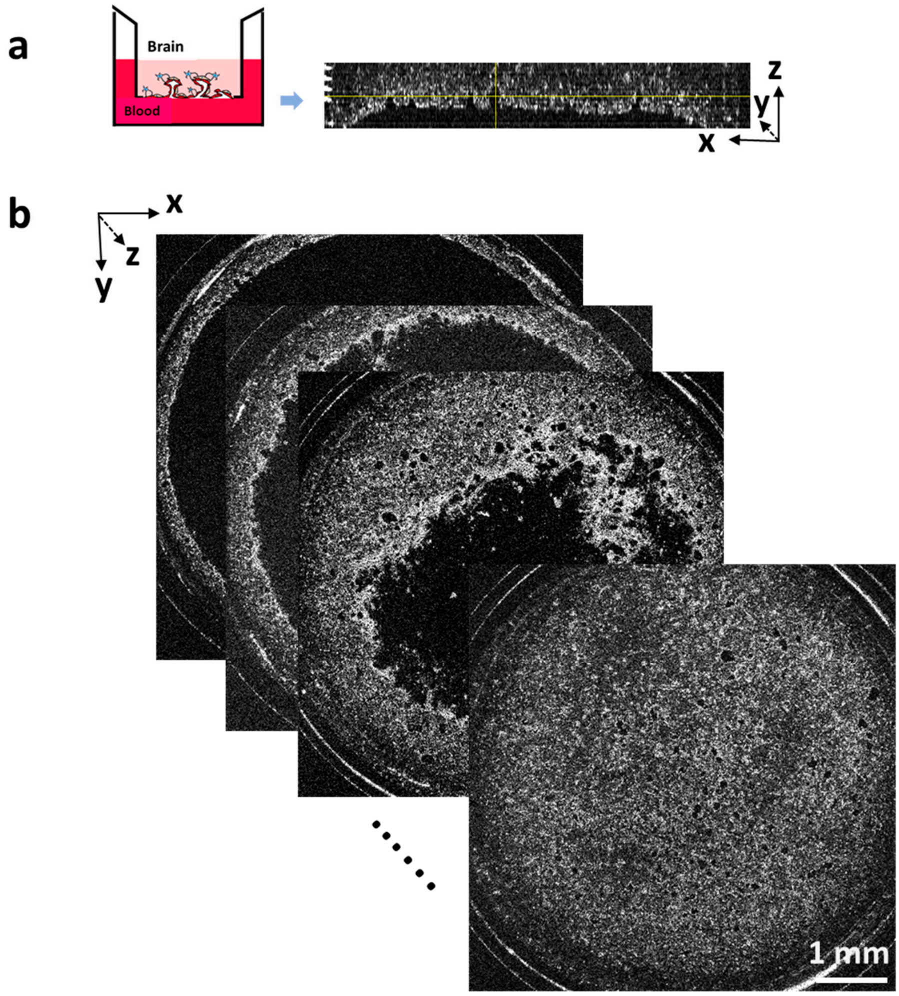

Figure S1. Front view of OCT image of x-z along y (a) and the stack images of x-y cross-sectional images along z of our BBB model (b).

a

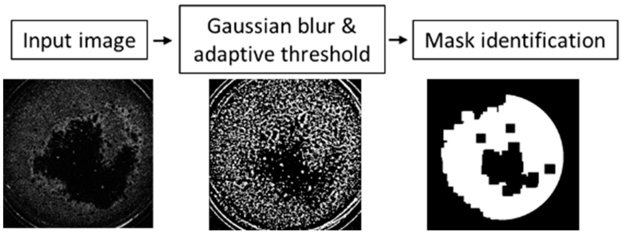

b

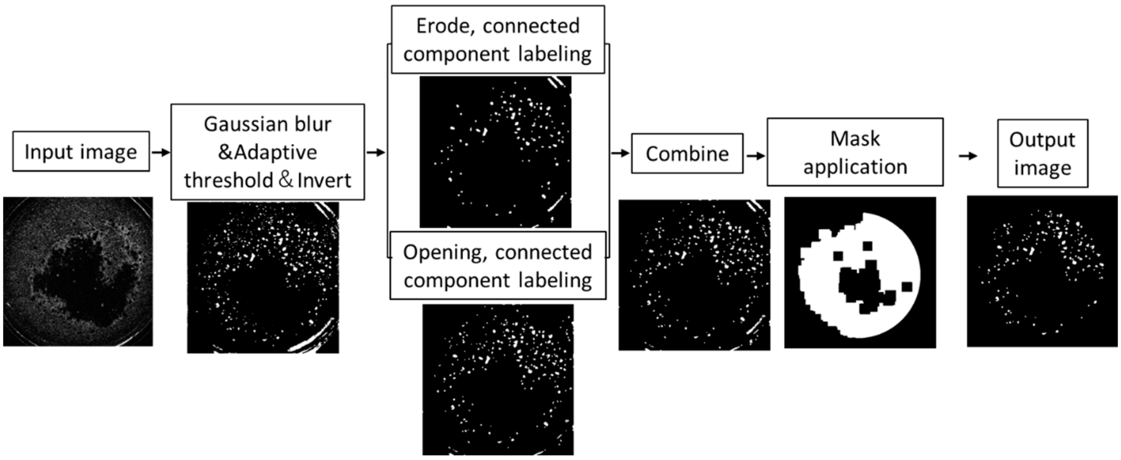

Figure S2. Flowchart of morphological imaging processing including the ROI mask generation (a), and the image processing to identify the pore-like morphology (b). Gaussian blur and adaptive threshold were performed on the contrast-enhanced images, the processed images were inverted for morphological erosion and opening to remove large or small dots from the images. The resultant images were combined and followed by filtering with ROI mask to remove untargeted area.

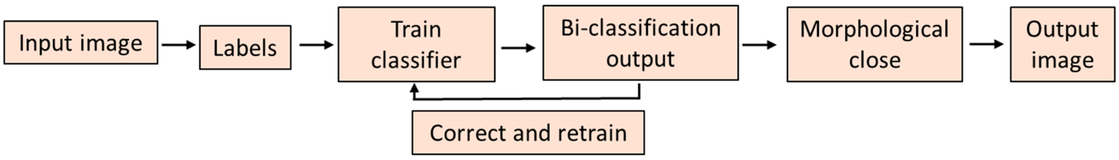

Figure S3. Workflow of image processing using RF-TWS. The original images were input into classifier followed by manual annotation of the pore-like and non-pore-like pixels to train the classifier. The output bi-classification images were post-processed with a morphological operator for final output images.

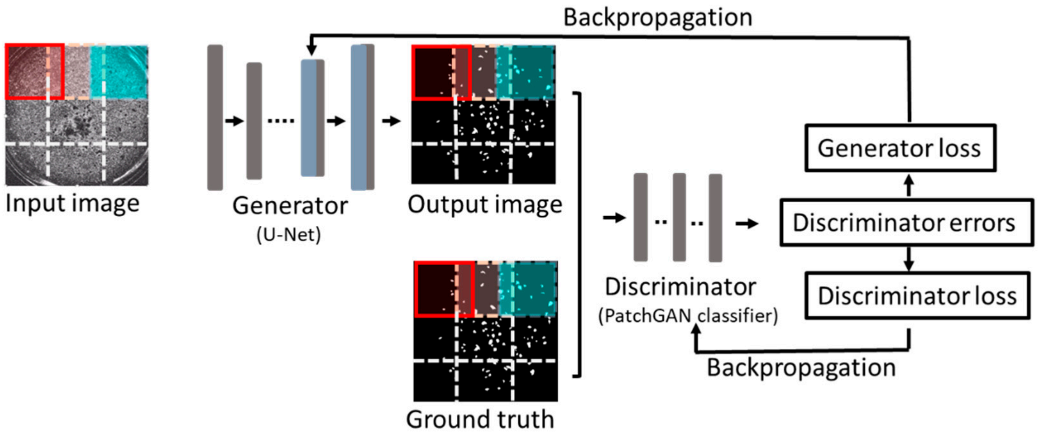

Figure S4. Deep learning using pix2pix cGAN architecture for image processing. Patch images were fed to the generator and translated to the target (output) images, while the discriminator distinguishes the generated image or the ground-truth image by assessing the discriminator errors.

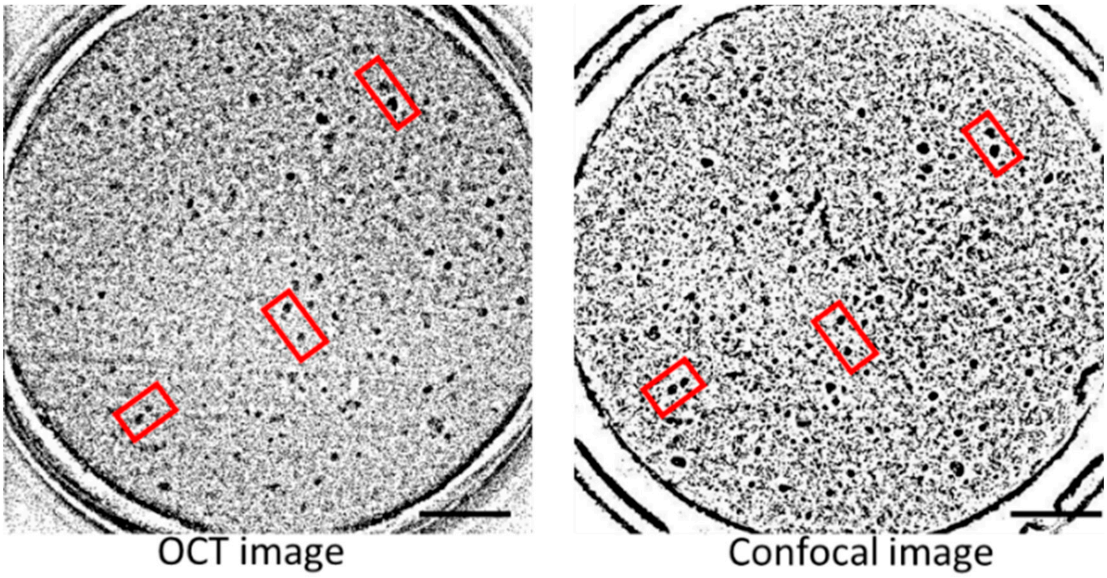

Figure S5. Comparison of the cross-sectional view images (sum slices and binarized) obtained by OCT and confocal microscopy (immunostaining with CD31). Red rectangles indicate representative, matched pore-like morphology in both. Scale bar = 1 mm.

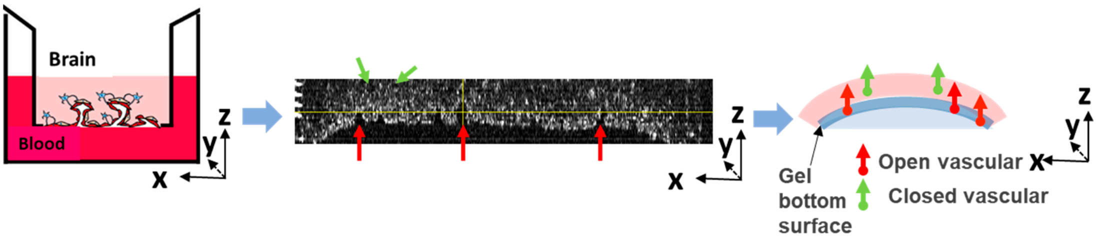

Figure S6. Front view of the open vascular (red arrow) and closed vascular structures (green arrow) shown in OCT images (middle) and illustrations (right).
